# Hyperlipidemia does not influence clinical outcome in arthroscopic treatment of femoroacetabular impingement syndrome

**DOI:** 10.1186/s13018-022-03290-3

**Published:** 2022-08-31

**Authors:** Fan Yang, Hong-Jie Huang, Zhu Zhang, Xin Zhang, Jian-Quan Wang

**Affiliations:** Department of Sports Medicine, Beijing Key Laboratory of Sports Injuries, Institute of Sports Medicine of Peking University, Peking University Third Hospital, 49 North Garden Rd., Haidian District, Beijing, 100191 People’s Republic of China

**Keywords:** Hyperlipidemia, Femoroacetabular impingement syndrome, Patient-reported outcomes

## Abstract

**Background:**

Cholesterol can trigger degenerative processes in the chondrocytes. The internal layer of the hip labral consists of cartilage-specific type II collagen-positive fibrocartilage. The purpose of this study was to compare outcomes after arthroscopy labral repair in FAIS patients with preoperative hyperlipidemia to a control group with no hyperlipidemia.

**Methods:**

Data were prospectively collected and retrospectively reviewed for FAIS patients who had arthroscopy 2019. Patients with hyperlipidemia were 1:1 propensity-score matched to patients without hyperlipidemia. Patient-reported outcomes (HOS-ADL, iHOT-12, mHHS), VAS scores, radiographic measures, performed procedures, complications, and revision surgery were compared and analyzed for both groups.

**Results:**

A total of 41 patients with hyperlipidemia and 41 patients without hyperlipidemia were found using propensity-score matching. When compared to preoperative levels, both groups demonstrated significant improvements in PROs and VAS scores at the final follow-up. Besides, there were no significant differences in preoperative scores and final outcome scores between the groups. Besides, there were no other differences in revision rate and the rate of meeting the PASS and MCID between the study and the control groups.

**Conclusion:**

It was demonstrated in this study that FAIS patients with hyperlipidemia can expect to experience similar good short-term patient-reported outcomes as compared with patients without hyperlipidemia.

**Level of evidence:**

Case-series study**;** Level of Evidence: Level III.

## Background

Over the last 20 years, acetabular labral tears have become increasingly recognized as a source of hip pain in the young, athletic patient population [1, 2]. Symptomatic labral tears were mainly secondary to femoroacetabular impingement syndrome (FAIS), which is considered a precursor to osteoarthritis (OA) [3]. Several RCTs studies had found that surgery was superior to nonoperative management for acetabular labral tears in younger patients [4, 5]. Hip arthroscopic surgery has become a common procedure for the correction of labral tears and can be considered to achieve the same long-term efficacy as open surgery [6]. The incidence of hip arthroscopy increased by 85% between 2011 and 2018 in the United States [7]. Though good clinical effects were achieved after surgery [8, 9]. Many demographic factors can influence the clinical outcomes of hip arthroscopy, such as higher age, higher BMI, and female patients [10–14], of which obesity demonstrated a twofold increased risk of conversion to total hip arthroplasty [15].

Metabolic syndrome (MetS) is generally considered a combination of obesity, hypertension, dyslipidemia, and impaired glucose tolerance [16]. The prevalence of MetS was over twofold higher in the OA population [17]. Perets et al. demonstrate that Hyperlipidemia indeed plays a role in the development of OA since an increased intake of cholesterol led to more severe OA features in the knee joints [17]. Metabolic disease may influence the healing processes of the sutured labrum, thus, leading to inferior clinical outcomes. Perets et al. reported that patients with diabetes mellitus demonstrated a non-statistically significant trend toward inferior outcomes in all patient-reported outcomes, visual analog scale scores, and satisfaction in FAIS patients [18]. However, few studies had focused on hyperlipidemia in FAIS patients treated with labral repair. Several studies suggested that cholesterol triggered degenerative processes in the cartilage by altered cholesterol homeostasis in the chondrocytes [19, 20]. The internal layer of the labral consists of cartilage-specific type II collagen-positive fibrocartilage [21].

This sparked speculation about whether hyperlipidemia in FAIS patients may influence the healing of sutured labrum, and then compromise the surgical outcomes. Therefore, the purpose of this study was to compare post-arthroscopy results in FAIS patients with hyperlipidemia to a control group with no hyperlipidemia.

## Materials and methods

Approval for the study was granted through the hospital review board IRB. A prospectively collected and retrospectively analysis was conducted of data from 2019. Symptoms, clinical signs and imaging findings must be present to diagnose FAIS [22]. Symptoms mainly including motion-related or position-related pain in the hip or groin. Clinical signs including a limited range of hip motion, flexion adduction internal rotation (FADIR) or flexion abduction external rotation (FABER) test. Indications for surgery were persistent pain, and failed conservative treatment after at least 3 months (physical therapy, oral anti-inflammatory drugs, and/or intra-articular injection). During the study period, a total of 290 hips underwent arthroscopic procedures, of which 208 hips had labral repair. Inclusion criteria included patients who underwent labral repair, aged from 18 to 50 years old, and with a minimum two years of follow-up. Exclusion criteria included: history of previous ipsilateral hip operation (21 hips), contralateral hip surgery during the follow-up time (12 hips), preoperative LCEA < 25° (16 hips), patients with diabetes mellitus (5 hips), moderate to advanced osteoarthritis (Tӧnnis grade ≥ 2) (3 hips), sacroiliac joint disease, and incomplete preoperative radiographs and medical record. Hyperlipidemia included one or both hypercholesterolemia and hypertriglyceridemia. Cut-off values used at our center for definitions of hypercholesterolemia and hypertriglyceridemia were total serum cholesterol ≥ 5.18 mmol/L and total serum triglyceride ≥ 1.7 mmol/L. The diagnosis of hyperlipidemia was conducted by one physician and it was carried out within one week before surgery. The study group was propensity-matched 1:1 to the FAIS patients without Hyperlipidemia.

Patient-reported outcomes (PROs), such as the Hip Outcome Score-Activities of Daily Living (HOS-ADL), Hip Outcome Score-Sport-Specific Subscale (HOS-SSS), and International Hip Outcome Tool 12-component form (iHOT-12), were used to evaluate the level of function included. Visual Analog Scale (VAS) was also provided for pain assessment. The VAS score for pain was evaluated on a scale of 0 (no pain) to 10 (extreme pain). Differences between preoperative and postoperative scores were calculated. The Minimal Clinically Important Difference (MCID) and Patient Acceptable Symptomatic State (PASS) were also calculated to determine meaningful outcome improvement. The published PASS cutoffs of PROs were used, 88.2 for HOS-ADL, 76.4 for HOS-SSS, 72.2 for iHOT-12, and 83.3 for mHHS. The MCID thresholds for HOS-ADL, HOS-SSS, iHOT-12, and mHHS were 9.7, 14.3, 13.9, and 9.2, respectively [23].

### Radiographic measurements

The patients underwent preoperative anteroposterior (AP) pelvis, 45° Dunn lateral radiography, and unilateral hip MRI. Radiographic measurements were performed using a picture archiving and communication system (PACS; GE Healthcare). The LCEA angle and Tӧnnis grade were measured on AP pelvis radiographs with an LCEA angle > 40° indicating pincer impingement. The alpha angle was measured on 45°Dunn lateral radiographs with an alpha angle > 55° indicating cam impingement. All MRI images were evaluated by one musculoskeletal radiologist and one senior author. Both observers were blinded to all clinical data of patients. They took two measurements one month apart to determine the reliability and produce clinically meaningful results. Intraobserver and interobserver reliability between the author and the musculoskeletal radiologist were calculated using the intraclass correlation coefficient (ICC). The ICC found that the intraobserver and interobserver reliability of gluteal tendinosis on MRI was greater than 0.80 for each parameter, indicating an acceptable level of reliability.

### Surgical procedures

All hip arthroscopies were performed by one senior author. The patient was placed in the modified supine position on standard hip traction (Smith & Nephew). The procedure began with fluoroscopic localization of the anterolateral (AL) portal, midanterior portal (MAP), and the proximal mid-anterior portal (PMAP) [24]. An interportal capsulotomy was performed. Pathology in the central compartment, including pincer deformity, labral injury, and chondrolabral injury, can be treated with these portals. Depending on the labral condition, labral tears were repaired with suture anchor fixation when possible. According to pre-operative and intra-operative imaging fluoroscopy, acetabular rim is resected or minimally burred to produce a bleeding bone bed for labral healing. Then the suture anchors are placed to reattach the labrum. After addressing pathology in the central compartment, the arthroscope was introduced into the peripheral compartment for decompression of the cam deformity by a high-speed burr (Smith & Nephew American). The capsular plication is routinely performed with approximately 3 to 4 interrupted stitches at the end of the procedure [25].

### Postoperative rehabilitation

All patients followed a well-standardized rehabilitation protocol under the direct supervision of our physiotherapist team as previously described [25]. Rehabilitation took an average of 4–5 months and was divided into four phases. Briefly, the first phase comprised isometric contractions and passive range-of-motion exercises. The second phase focused on maintaining a regular gait and restoring a full range of motion. The third phase was about regaining lower extremity strength as well as normal functional activities. The final phase focused on resuming pre-injury higher-level activities.

### Statistical analysis

An a priori power analysis was performed to determine the number of patients required to detect a statistical difference. Based on the assumption that a mean difference of 8.3 points in follow-up mHHS between groups is clinically important. The sample size was determined to be 17 patients per group [26], using alpha at 0.05, and beta at 0.2 (80% power). A 1:1 propensity-score match based on age, gender, body mass index (BMI), Tӧnnis grade, and follow-up time was performed to control for potential confounding variables in the study group and the control group. The Shapiro–Wilk test was used to examine the data to determine whether all parametric statistical assumptions were satisfied. We used a 2-tailed unpaired Student t-test to compare continuous demographic data between the two groups. Nonparametric testing was used for analysis in cases where parametric statistical assumptions were violated. The 2-tailed paired Student t-test was used to compare pre-and post-operative PROs. The chi-square test or Fisher exact test was used to compare categorical variables between the two groups. SPSS version 26 (IBM, Armonk, NY) was used for all statistical analyses. P-values less than 0.05 were considered statistically significant.

## Results

### Characteristics of the patients and performed procedures

Both the inclusion and exclusion criteria were met in 151 patients, 135 patients (89%) had a minimum 2-year follow-up, of which 49 patients had Hyperlipidemia. Propensity-score matching yielded 42 patients in the study group and 42 patients in the study group. There were no significant differences detected in age, sex, BMI, Tӧnnis grade, or follow-up time after matching the two groups. The demographic and radiographic data of all patients are presented in Table 1.

**Table 1:** Characteristics of the patients

Category	Study group	Control group	*P*-value
No. of hips	42	42	
Age, yr	39.1 ± 9.8	39.4 ± 8.9	0.617
BMI, kg/m2	24.1 ± 3.0	23.5 ± 3.0	0.356
Sex, n (%)			0.827
Female	20 (47.6)	19 (45.2)	
Male	22 (52.4)	23 (54.8)	
Follow-up time, m	36.1 ± 3.2	35.3 ± 3.6	0.714
Cholesterol	5.2 ± 0.71	4.2 ± 0.53	**< 0.001**
Triglyceride	1.9 ± 0.98	1.1 ± 0.4	**< 0.001**
LCEA preoperative	35.5 ± 5.5	35.4 ± 5.6	0.934
LCEA postoperative	29.2 ± 4.5	29.6 ± 5.1	0.728
Alpha preoperative	63.8 ± 9.2	63.7 ± 8.1	0.945
Alpha postoperative	42.5 ± 5.0	41.4 ± 4.2	0.273

Bold value indicates statistical significance

Values are given as mean ± SD

*BMI* Body mass index

Overall, all patients had a labral repair, and the majority of the study population femoral osteoplasty (98.7%), and acetabular rim trimming (76.8%). There were no significant differences in intraoperative variables between the two groups.

### Patient-reported outcomes

**Fig. 1:**
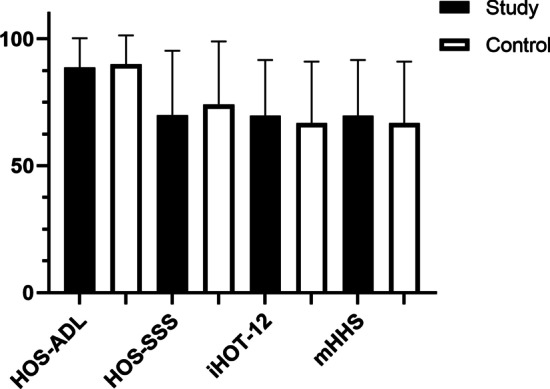
Box plot showing results of patient-reported outcome scores for the study and control groups. HOS-ADL, Hip Outcome Scored-Activities of Daily Living; HOS-SSS, Hip Outcome Score-Sport-Specific Subscale; IHOT-12, International Hip Outcome Tool 12-component form; mHHS, modified Harris Hip Score

Both groups demonstrated significant improvements in PROs at the final follow-up compared with preoperative levels. (*p* < 0.001 for all) (Table 2). Besides, there were no significant differences in preoperative scores, and final outcome scores, between the two groups (Fig. 1). The effect size (ES) of postoperative PROs of HOS-ADL, HOS-SSS, iHOT-12, mHHS were 0.120, 0.168, 0.125, 0.268, respectively. Besides, there were no differences in the rate of meeting the PASS and MCID between the study group and the control group (Table 3).

**Table 2:** Outcomes of hip arthroscopic

	Study group	Control group	*P*-value
*HOS-ADL*			
Preoperative	72.3 ± 26.0	72.0 ± 24.3	0.949
Postoperative	88.7 ± 11.6	90.1 ± 11.3	0.590
*P*-value (pre-post)	< **0.001**	**0.001**	
*HOS-SSS*			
Preoperative	51.0 ± 28.5	52.8 ± 29.8	0.775
Postoperative	70.0 ± 25.4	74.2 ± 24.8	0.452
*P*-value (pre-post)	< **0.001**	< **0.001**	
*iHOT-12*			
Preoperative	45.3 ± 20.2	44.5 ± 24.1	0.868
Postoperative	69.7 ± 22.0	66.8 ± 24.3	0.574
*P*-value (pre-post)	< **0.001**	< **0.001**	
*mHHS*			
Preoperative	56.4 ± 19.2	57.7 ± 17.7	0.755
Postoperative	76.1 ± 16.2	79.9 ± 11.8	0.225
*P*-value (pre-post)	< **0.001**	< **0.001**	
*VAS*			
Preoperative	6.0 ± 2.5	6.3 ± 2.2	0.542
Postoperative	3.2 ± 2.7	3.1 ± 2.6	0.902
*P*-value (pre-post)	< **0.001**	< **0.001**	

Data are reported as mean ± SD

*HOS-ADL* Hip outcome scored-activities of daily living, *HOS-SSS* Hip outcome score-sport-specific subscale, *IHOT-12* International hip outcome tool 12-component form, *mHHS* modified Harris hip score, *VAS* Visual analog score. Bold value indicates statistical significance

**Table 3:** Rates of MCID and PASS

	Study group	Control group	*P*-value
*MCID*			
HOS-ADL	27 (64.3)	31 (73.8)	0.513
HOS-SSS	24 (57.1)	25 (59.5)	0.825
iHOT-12	26 (61.9)	26 (61.9)	> 0.999
mHHS	29 (69)	33 (78.6)	0.321
*PASS*			
HOS-ADL	19 (45.2)	22 (52.4)	0.345
HOS-SSS	18 (42.9)	21 (50.0)	0.512
iHOT-12	24 (57.1)	23 (54.8)	0.826
mHHS	20 (47.6)	24 (57.1)	0.382

Data are shown as n (%)

*HOS-ADL* Hip outcome scored-activities of daily living, *HOS-SSS* Hip outcome score-sport-specific subscale, *IHOT-12* International hip outcome tool 12-component form, *mHHS* modified Harris hip score

### Complications and secondary surgery

There were no significant differences between the groups concerning complications. During the final follow-up, a total of three hips (2.4%) underwent revision hip arthroscopy. Two patients in the study group had residual cam deformity and gradual onset of symptoms. One patient underwent revision due to subspine impingement (SSI). No patient required conversion to THA in both groups.

## Discussion

The main findings of this study were that a majority of FAIS patients with or without hyperlipidemia can obtain clinically significant outcomes with the arthroscopic repair of the labrum followed by a minimum follow-up of two years. Besides, there were no differences in the revision rate and the rate of meeting the PASS and MCID between the study group and the control group.

Labral repair was considered the gold standard in hip arthroscopy treatment of labral tears. In hip arthroscopy treatment of labral pathology, two technical aspects vary greatly between specialists, Labral debridement or excision has historically been used to treat acetabular labral tears, which reduces pain for patients [27]. However, increasing evidence suggests that labral repair or refixation restores the suction seal of the normal labrum, reduces femoral head translation, and reduces acetabular contact stress [28]. There is rapidly growing basic science evidence in support of capsular repair to restore more normal hip biomechanics after surgery [29, 30]. A randomized controlled trial revealed that arthroscopic treatment of FAIS with labral repair in female patients resulted in superior improvement in hip functional outcomes compared with labral debridement [31]. A recent systematic review also showed that when comparing labral repair with labral debridement, labral repair renders a reduced risk of reoperation [32].

Impaired lipid metabolism has been recognized as an important factor in OA development [33]. A high-fat diet is an aggravating factor worsening synovial membrane inflammation during OA [34]. Several studies suggested that cholesterol triggered degenerative processes in the cartilage by altered cholesterol homeostasis in the chondrocytes. The expression of genes regulating cholesterol efflux in human OA chondrocytes is impaired, causing lipid droplets to accumulate toxically [19, 35]. Studies also have demonstrated an association between OA pathogenesis and SREBP-2, a gene important in cholesterol homeostasis [20]. Though the peripheral aspect of the acetabular labrum consists of dense connective tissue, the internal layer consists of type II collagen-positive fibrocartilage, especially on the articular surface, there is a layer of collagen fibrils beneath the superficial thin fibrils. Lipid toxically may influence the chondrocytes of the labrum, thus affecting the healing of the labral.

Previous studies had confirmed there is an association between hyperlipidemia and osteochondral lesions. Frey et.al observed an increased risk of osteoarthritis of the hand (HOA) in patients with hyperlipidaemia (OR 1.37). Though most HOA patients were elderly. Furthermore, they observed higher relative risk in younger patients, with the highest OR of 1.72 in patients aged 29–49 years[36]. Davies et.al found cholesterol (OR 1.84) and triglycerides (OR 8.4) are associated with the development of new bone marrow lesions in asymptomatic middle-aged women. They postulated that reducing serum lipids may have a role in reducing the burden of knee OA [37]. Pan et.al reported that hypertriglyceridemia (relative risk ratio: 1.75) and low HDL (relative risk ratio: 1.67) were associated with moderate pain in OA patients [38]. However, this study showed that patients with hyperlipidemia can expect to experience similar good short-term patients-reported outcomes. Long-term follow-up studies are needed to confirm the results.

This study has certain limitations. First, because the study group only included patients who had FAIS surgery, the results may not apply to an average FAIS patient. Second, because this was a retrospective study, there was an inherent bias. Despite propensity-score matching being used to control for potential confounding variables, additional confounding variables could have influenced our results. Third, imaging to follow up is needed to assess the quality of labral healing in two groups.

## Conclusion

This study demonstrated that FAIS patients with hyperlipidemia can expect to experience similar good short-term patients-reported outcomes as compared with patients without hyperlipidemia.

## Data Availability

The datasets used and/or analyzed during the current study are available from the corresponding author on reasonable request.

## Abbreviations

FAIS: Femoroacetabular impingement syndrome
MRI: Magnetic resonance imaging

## References

[1] Martin SD, Abraham PF, Varady NH, Nazal MR, Conaway W, Quinlan NJ, Alpaugh K (2021). Hip arthroscopy versus physical therapy for the treatment of symptomatic acetabular labral tears in patients older than 40 years: a randomized controlled trial. Am J Sports Med.

[2] Bedi A, Chen N, Robertson W, Kelly BT (2008). The management of labral tears and femoroacetabular impingement of the hip in the young, active patient. Arthroscopy.

[3] Beck M, Kalhor M, Leunig M, Ganz R (2005). Hip morphology influences the pattern of damage to the acetabular cartilage: femoroacetabular impingement as a cause of early osteoarthritis of the hip. J Bone Joint Surg Br.

[4] Impellizzeri FM, Jones DM, Griffin D, Harris-Hayes M, Thorborg K, Crossley KM, Reiman MP, Scholes MJ, Ageberg E, Agricola R (2020). Patient-reported outcome measures for hip-related pain: a review of the available evidence and a consensus statement from the International Hip-related pain research network, Zurich 2018. Br J Sports Med.

[5] Palmer AJR, Ayyar Gupta V, Fernquest S, Rombach I, Dutton SJ, Mansour R, Wood S, Khanduja V, Pollard TCB, McCaskie AW (2019). Arthroscopic hip surgery compared with physiotherapy and activity modification for the treatment of symptomatic femoroacetabular impingement: multicentre randomised controlled trial. BMJ (Clinical Research ed).

[6] Migliorini F, Liu Y, Eschweiler J, Baroncini A, Tingart M, Maffulli N (2022). Increased range of motion but otherwise similar clinical outcome of arthroscopy over open osteoplasty for femoroacetabular impingement at midterm follow-up: a systematic review. Surg J R Coll Surg Edinb Irel.

[7] Zusmanovich M, Haselman W, Serrano B, Banffy M (2022). The Incidence of hip arthroscopy in patients with femoroacetabular impingement syndrome and labral pathology increased by 85% between 2011 and 2018 in the United States. Arthroscopy.

[8] Migliorini F, Liu Y, Catalano G, Trivellas A, Eschweiler J, Tingart M, Maffulli N (2021). Medium-term results of arthroscopic treatment for femoroacetabular impingement. Br Med Bull.

[9] Migliorini F, Baroncini A, Eschweiler J, Knobe M, Tingart M, Maffulli N (2021). Return to sport after arthroscopic surgery for femoroacetabular impingement. Surg J R Coll Surg Edinb Irel.

[10] Seijas R, Barastegui D, López-de-Celis C, Montaña F, Cuscó X, Alentorn-Geli E, Samitier-Solis G, Cugat R (2021). Preoperative risk factors in hip arthroscopy. Knee Surg Sports Traumatol Arthrosc.

[11] Bloom DA, Fried JW, Bi AS, Kaplan DJ, Chintalapudi N, Youm T (2020). Age-associated pathology and functional outcomes after hip arthroscopy in female patients: analysis with 2-year follow-up. Am J Sports Med.

[12] Marland JD, Horton BS, West HS, Wylie JD (2022). Association of radiographic markers of hip instability and worse outcomes 2 to 4 years after hip arthroscopy for femoroacetabular impingement in female patients. Am J Sports Med.

[13] Frank RM, Lee S, Bush-Joseph CA, Salata MJ, Mather RC, Nho SJ (2016). Outcomes for hip arthroscopy according to sex and age: a comparative matched-group analysis. J Bone Joint Surg Am.

[14] Migliorini F, Maffulli N, Baroncini A, Eschweiler J, Tingart M, Betsch M (2022). Revision surgery and progression to total hip arthroplasty after surgical correction of femoroacetabular impingement: a systematic review. Am J Sports Med.

[15] Perets I, Rybalko D, Chaharbakhshi EO, Mu BH, Chen AW, Domb BG (2018). Minimum five-year outcomes of hip arthroscopy for the treatment of femoroacetabular impingement and labral tears in patients with obesity: a match-controlled study. J Bone Jt Surg Am.

[16] Huang PL (2009). A comprehensive definition for metabolic syndrome. Dis Model Mech.

[17] Puenpatom RA, Victor TW (2009). Increased prevalence of metabolic syndrome in individuals with osteoarthritis: an analysis of NHANES III data. Postgrad Med.

[18] Perets I, Chaharbakhshi EO, Barkay G, Mu BH, Lall AC, Domb BG (2021). Diabetes mellitus is not a negative prognostic factor for patients undergoing hip arthroscopy. Orthopedics.

[19] Tsezou A, Iliopoulos D, Malizos KN, Simopoulou T (2010). Impaired expression of genes regulating cholesterol efflux in human osteoarthritic chondrocytes. J Orthop Res.

[20] Kostopoulou F, Gkretsi V, Malizos KN, Iliopoulos D, Oikonomou P, Poultsides L, Tsezou A (2012). Central role of SREBP-2 in the pathogenesis of osteoarthritis. PLoS One.

[21] Petersen W, Petersen F, Tillmann B (2003). Structure and vascularization of the acetabular labrum with regard to the pathogenesis and healing of labral lesions. Arch Orthop Trauma Surg.

[22] Griffin DR, Dickenson EJ, O'Donnell J, Agricola R, Awan T, Beck M, Clohisy JC, Dijkstra HP, Falvey E, Gimpel M (2016). The Warwick Agreement on femoroacetabular impingement syndrome (FAI syndrome): an international consensus statement. Br J Sports Med.

[23] Nwachukwu BU, Beck EC, Kunze KN, Chahla J, Rasio J, Nho SJ (2020). Defining the clinically meaningful outcomes for arthroscopic treatment of femoroacetabular impingement syndrome at minimum 5-year follow-up. Am J Sports Med.

[24] Philippon MJ, Schenker ML (2006). Arthroscopy for the treatment of femoroacetabular impingement in the athlete. Clin Sports Med.

[25] Yang F, Mamtimin M, Duan YP, Sun H, Xu Y, Zhang X, Zheng XY, Fan JL, Huang HJ, Wang JQ (2021). Volume of gluteus maximus and minimus increases after hip arthroscopy for femoroacetabular impingement syndrome. Arthroscopy.

[26] Jimenez AE, Monahan PF, Miecznikowski KB, Saks BR, Ankem HK, Sabetian PW, Lall AC, Domb BG (2021). Achieving successful outcomes in high-level athletes with borderline hip dysplasia undergoing hip arthroscopy with capsular plication and labral preservation: a propensity-matched controlled study. Am J Sports Med.

[27] Shindle MK, Voos JE, Nho SJ, Heyworth BE, Kelly BT (2008). Arthroscopic management of labral tears in the hip. J Bone Joint Surg Am.

[28] Philippon MJ, Nepple JJ, Campbell KJ, Dornan GJ, Jansson KS, LaPrade RF, Wijdicks CA (2014). The hip fluid seal–Part I: the effect of an acetabular labral tear, repair, resection, and reconstruction on hip fluid pressurization. Knee Surg Sports Traumatol Arthrosc.

[29] Philippon MJ, Trindade CAC, Goldsmith MT, Rasmussen MT, Saroki AJ, Løken S, LaPrade RF (2017). Biomechanical assessment of hip capsular repair and reconstruction procedures using a 6 degrees of freedom robotic system. Am J Sports Med.

[30] Martin HD, Khoury AN, Schröder R, Johnson E, Gómez-Hoyos J, Campos S, Palmer IJ (2017). Contribution of the pubofemoral ligament to hip stability: a biomechanical study. Arthroscopy.

[31] Krych AJ, Thompson M, Knutson Z, Scoon J, Coleman SH (2013). Arthroscopic labral repair versus selective labral debridement in female patients with femoroacetabular impingement: a prospective randomized study. Arthroscopy.

[32] Riff AJ, Kunze KN, Movassaghi K, Hijji F, Beck EC, Harris JD, Nho SJ (2019). Systematic review of hip arthroscopy for femoroacetabular impingement: the importance of labral repair and capsular closure. Arthroscopy.

[33] Gierman LM, Kühnast S, Koudijs A, Pieterman EJ, Kloppenburg M, van Osch GJVM, Stojanovic-Susulic V, Huizinga TWJ, Princen HMG, Zuurmond AM (2014). Osteoarthritis development is induced by increased dietary cholesterol and can be inhibited by atorvastatin in APOE*3Leiden.CETP mice–a translational model for atherosclerosis. Ann Rheum Dis.

[34] Larrañaga-Vera A, Lamuedra A, Pérez-Baos S, Prieto-Potin I, Peña L, Herrero-Beaumont G, Largo R (2017). Increased synovial lipodystrophy induced by high fat diet aggravates synovitis in experimental osteoarthritis. Arthritis Res Ther.

[35] Tabas I (2002). Consequences of cellular cholesterol accumulation: basic concepts and physiological implications. J Clin Investig.

[36] Frey N, Hügle T, Jick SS, Meier CR, Spoendlin J (2017). Hyperlipidaemia and incident osteoarthritis of the hand: a population-based case-control study. Osteoarthr Cartil.

[37] Davies-Tuck ML, Hanna F, Davis SR, Bell RJ, Davison SL, Wluka AE, Adams J, Cicuttini FM (2009). Total cholesterol and triglycerides are associated with the development of new bone marrow lesions in asymptomatic middle-aged women—a prospective cohort study. Arthritis Res Ther.

[38] Pan F, Tian J, Cicuttini F, Jones G (2020). Metabolic syndrome and trajectory of knee pain in older adults. Osteoarthr Cartil.

